# Environmental impacts on intraspecific variation in *Ambrosia artemisiifolia* genome size in Slovakia, Central Europe

**DOI:** 10.1007/s11356-024-33410-x

**Published:** 2024-05-02

**Authors:** Michal Hrabovský, Silvia Kubalová, Karol Mičieta, Jana Ščevková

**Affiliations:** Department of Botany, Faculty of Natural Sciences, Comenius University, Révová 39, 811 02 Bratislava, Slovakia

**Keywords:** Ragweed, Absolute DNA amount, Flow cytometry, Climatic factors, Geographical variables

## Abstract

The quantity of DNA in angiosperms exhibits variation attributed to many external influences, such as environmental factors, geographical features, or stress factors, which exert constant selection pressure on organisms. Since invasive species possess adaptive capabilities to acclimate to novel environmental conditions, ragweed (*Ambrosia artemisiifolia* L.) was chosen as a subject for investigating their influence on genome size variation. Slovakia has diverse climatic conditions, suitable for testing the hypothesis that air temperature and precipitation, the main limiting factors of ragweed occurrence, would also have an impact on its genome size. Our results using flow cytometry confirmed this hypothesis and also found a significant association with geographical features such as latitude, altitude, and longitude. We can conclude that plants growing in colder environments farther from oceanic influences exhibit smaller DNA amounts, while optimal growth conditions result in a greater variability in genome size, reflecting the diminished effect of selection pressure.

**Supplementary Information:**

The online version contains supplementary material available at 10.1007/s11356-024-33410-x.

## Introduction

Genome size, a karyological characteristic denoting the amount of DNA within a cell (Greilhuber et al. 2005), exhibits significant variation in land plants and is known to exert a notable influence on their evolutionary trajectory (Gregory and Hebert 1999; Gregory 2005; Knight et al. 2005; Lysak et al. 2009; Pellicer et al. 2018). Interspecific genome size variation is well known due to many phylogenetic and taxonomic investigations (e.g., Kron et al. 2007; Kolář et al. 2009, 2013; Marhold et al. 2010; Španiel et al. 2011; Dirkse et al. 2014; Abbasi-Karin et al. 2022). In contrast, the intraspecific variation among populations or individuals was revealed for a small number of species (e.g., *Festuca pallens*, Šmarda and Bureš 2006; Šmarda et al. 2008, 2010, *Lythrum salicaria*, Kubátová et al. 2008; *Phragmites australis*, Meyerson et al. 2016; Pyšek et al. 2020, or *Euphrasia arctica*, Becher et al. 2021). The plant genome size can be linked for various species or taxonomic groups with life cycle and life form (Bennett 1972; Albach and Greilhuber 2004; Hidalgo et al. 2015; Shao et al. 2021; Carta et al. 2022), growth form (Ohri 2005; Dušková et al. 2010; Trávníček et al. 2019), climatic factors (Bennett et al. 1982; Bennett 1987; Wakamiya et al. 1993; Caceres et al. 1998; Carta and Peruzzi 2016), geographical features (Levin and Funderburg 1979; Rayburn 1990; Bottini et al. 2000; Knight et al. 2005; Meyerson et al. 2016; Bureš et al. 2024), invasiveness (Bennett et al. 1998; Grotkopp et al. 2004; Kubešová et al. 2010; Pyšek et al. 2020), stress factors or pollution (Madlung and Comai 2004; Vidic et al. 2009; Meyerson et al. 2020), metabolic resources such as phosphorus and nitrogen (Hessen et al. 2010; Guignard et al. 2016), or phylogenetic age (Farah et al. 2018; Hoang et al. 2020). Despite much evidence of adaptive selection, genetic drift cannot be excluded from the genome size distribution (Blommaert 2020).

On a broad scale encompassing both geographical and environmental factors, correlations between genome size and these variables have been predominantly observed in native perennial species (e.g., Wakamiya et al. 1993; Bottini et al. 2000; Mahdavi and Karimzadeh 2010; Enke et al. 2011; Yotoko et al. 2011; Carta and Peruzzi 2016; Meyerson et al. 2016; Sayadi et al. 2022). On the other hand, annual plant species can exhibit more rapid evolutionary adaptation (Franks et al. 2007; Osnato 2022) or undergo faster natural selection (Larios et al. 2014) in response to environmental changes than perennials. However, these evolutionary mechanisms may not be effective in small populations subject to genetic drift (Andrews 2010), whereas gene flow in large populations can potentially counteract selective pressures (Sork 2015). The alien invasive species often have smaller genomes than their non-invasive relatives (Bennett et al. 1998; Grotkopp et al. 2004; Kubešová et al. 2010; Pyšek et al. 2020), and in a changing world, they can adapt better to changing climatic conditions than their relatives with large genomes (Grime 1998; Knight and Ackerly 2002; Vidic et al. 2009). They can evolve rapidly in response to selection pressures in the new environment (Dlugosch and Parker 2008; Zenni et al. 2017).

For the study of the adaptation processes on the level of the genome size, the common ragweed (*Ambrosia artemisiifolia* L.) belonging to the Asteraceae family is a suitable model organism, due to its following characteristics. It has a high spread potential in Europe (Lambdon et al. 2008), where it was introduced from North America and naturalized more than 70–150 years ago, depending on the region (Dessaint et al. 2005; Hrabovský et al. 2016; Skálová et al. 2017; Pinke et al. 2019). Climate change, in conjuction with anthropogenic influences, can facilitate the spread of invasive thermophilic ragweed to colder regions (Cunze et al. 2013; Rasmussen et al. 2017; Skálová et al. 2017; Mang et al. 2018; Lemke et al. 2021; Liu et al. 2021). As an annual plant, it produces seeds within one growing season, 4 to 6 months after germination (Kazinczi et al. 2008). It is known that the range of its occurrence is limited by air temperature and precipitation (Gentili et al. 2019); however, the species has demonstrated the ability to adapt in mountainous regions recently (Kochjarová et al. 2023). The large invasive ragweed populations exhibit high levels of genetic diversity attributable to complete outcrossing (Genton et al. 2005; Chun et al. 2010; Meyer et al. 2017), while the related perennial *Ambrosia psilostachya* shows clonality and low genetic diversity (Karrer et al. 2023). Some intraspecific variation in ragweed genome size is documented as its DNA amount varies from 2.08 to 2.27 pg/2C (Kubešová et al. 2010; Bai et al. 2012; Battlay et al. 2023). However, this variability has not been linked to environmental factors that are known to affect genome size selection (Knight and Ackerly 2002; Knight et al. 2005; Bureš et al. 2024).

The European continent is characterized by diverse ecological conditions and is therefore divided into a number of different environmental zones (Metzger et al. 2005). The territory of Slovakia extends into two zones, the lowland Pannonian and mountain Carpathian, thus providing the opportunity to investigate the effect of altitude, temperature, and precipitation gradients on the genome size variation. Ragweed was only found in the warm Pannonian flora region until 2000 (Jehlík 1998). After 2000, mountainous areas showed an increase in ragweed occurrence (Hrabovský and Mičieta 2018; Kochjarová et al. 2023).

We assume, that the genome size of annual alien species may exhibit variation within a limited geographical region, where diverse environmental factors can act upon naturalized populations over a specific period. This study endeavors to address the following inquiries: (i) To what extent does intraspecific variation exist in the DNA content of naturalized alien species? (ii) Which environmental factors are associated with genome size variability? (iii) Is there evidence to suggest that environmental factors contribute to variability in genome size during or after naturalization, possibly as a result of climate change?

## Materials and methods

### Field sampling

**Fig. 1:**
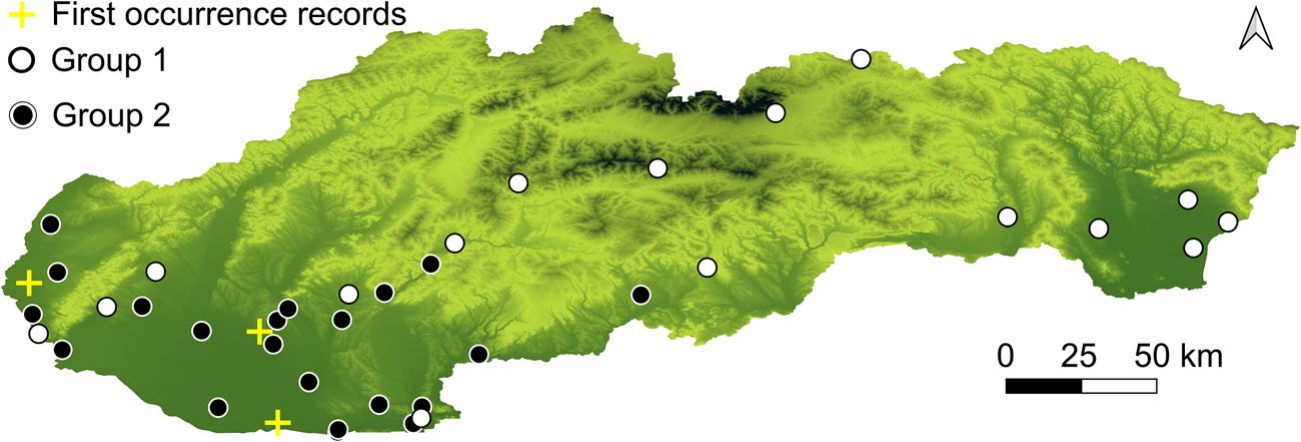
The study area with selected populations for measurement. First-occurrence records were measured from herbarium specimens. After measurements, the populations with a median genome size smaller than 2.1 pg/2C were labelled as group 1, and the other populations with a genome size larger than 2.1 pg/2C were labelled as group 2

Plant material was collected from 37 naturalized populations of invasive annual species *Ambrosia artemisiifolia* from southern Slovakia in the northernmost regions of the Pannonian Basin and adjacent Carpathian area, where the populations have been expanding since the late twentieth century (Jehlík 1998) (Fig. 1). Populations of the ragweed were selected accidentally from the Vienna Basin, Danubian Lowland, Western Carpathians, and the Eastern Slovak Lowland. The abundance of common ragweed in the examined area influenced the choice of populations, as more populations were selected from the west of the study area, where ragweed is widespread (Hrabovský et al. 2016). The populations can be classified as Carpathian or Pannonian based on the phytogeographic division of the area (Futák 1984). Anthropogenic habitats, including roadsides, arable land, railways, and ruderal habitats, were among the diverse habitats sampled. A distal leaf of at least five individuals was collected from each population. Planar-colline levels at the interface of the Pannonian Basin and the Western Carpathians exhibit distinct variations in elevations, temperatures, and precipitation. The majority of the examined region exhibits an elevation range of 110 to 330 m a. s. l., with localized exceptions where altitude may fall below 100 m or exceed 800 m. According to WorldClim datasets (Fick and Hijmans 2017), the mean annual temperature in the region fluctuates between 10 and 12.2 °C, while the average annual precipitation totals for the previous decade are reported to range from 600 to 700 mm.

### Genome size estimation

To estimate the 2C value, the methodical recommendations of Sliwinska et al. (2022) were followed, except for the direct analysis of fresh material. Fresh leaves from flowering plants were dried in silica gel. Plant material was collected during the 1-week period in August–September 2022. Leaf tissues were analyzed sequentially with an established standard, and the ratios of their G_0/1_ peak positions were recorded. As the established standard for the samples, leaves of *Bellis perennis* were chosen (2C = 3.159 pg; Temsch et al. 2022). The nuclei were isolated using a commercial reagent kit, Cystain PI OxProtect (Sysmex, United Kingdom), from a sample and co-chopped standard, and stained with propidium iodide (PI). The analyses were performed using a Partec CyFlow Ploidy Analyzer, equipped with a green laser (532 nm). At least 5000 nuclei were analyzed from each sample at least three times on different days. Samples that exhibited a coefficient of variation (CV) greater than 3% were excluded from the analysis, and additional samples were analyzed to ensure that data from at least five individuals per population were obtained (Table S1).

This study was preceded by verification of intrapopulation variability in three populations, where 25 2-week-old seedlings grown under the same conditions were analyzed from fresh leaves using the same procedure as above (Table S2, Fig. S1). We also estimated the genome size of the first ragweed individuals introduced to the study area (1949–1956) and later occuring populations (1957–1984) from herbarium specimens. According to Viruel et al. (2019), it is difficult to obtain results from old specimens, but it is possible to estimate a DNA amount even from 100-year-old specimens (Michalová et al. 2024). A part of a young green leaf (0.5 × 0.5 cm) was macerated for 30 min in a Cystain PI OxProtect nuclei extract solution. Then it was co-chopped with the standard and stained with PI. The quality of the herbarium item determines the degree of success. The samples with a CV greater than 7% were not included in this study.

The conformity of the results with prior estimations, which were based on the standards calibrated from *Oryza sativa* ssp. *japonica* cv. Nipponbare with an outdated value of 2C = 0.910 pg/2C, could be attained by recalculating the published data based on the current value of Nipponbare rice, which is 2C = 0.778 pg/2C as stated by Temsch et al. (2022).

### Climatic variables

For each population at each location, the following environmental factors were obtained from WorldClim (Fick and Hijmans 2017) using GIS software (QGIS 3.22.3): mean annual air temperature (*T*_mean_), mean May–October air temperature relating to the ragweed vegetation season (*T*_mean_ May–October), mean December − March air temperature relating to the dormancy period (*T*_mean_ December–March), annual precipitation totals (P), seasonal May − October precipitation totals (P May–October), and December − March precipitation totals (P December–March). These factors were extracted for both the historical period of ragweed naturalization in the study area (1970 − 2000) and the current period (2020–2021), air temperature in °C, and precipitation totals in mm. The air temperature and precipitation are the most notable indicators of continentality in the study area (Labudová et al. 2013; Vilček et al. 2016). The influence of continentality on ragweed genome size was presented through the *T*_mean_ and P May–October of the current period.

### Data analysis

**Fig. 2:**
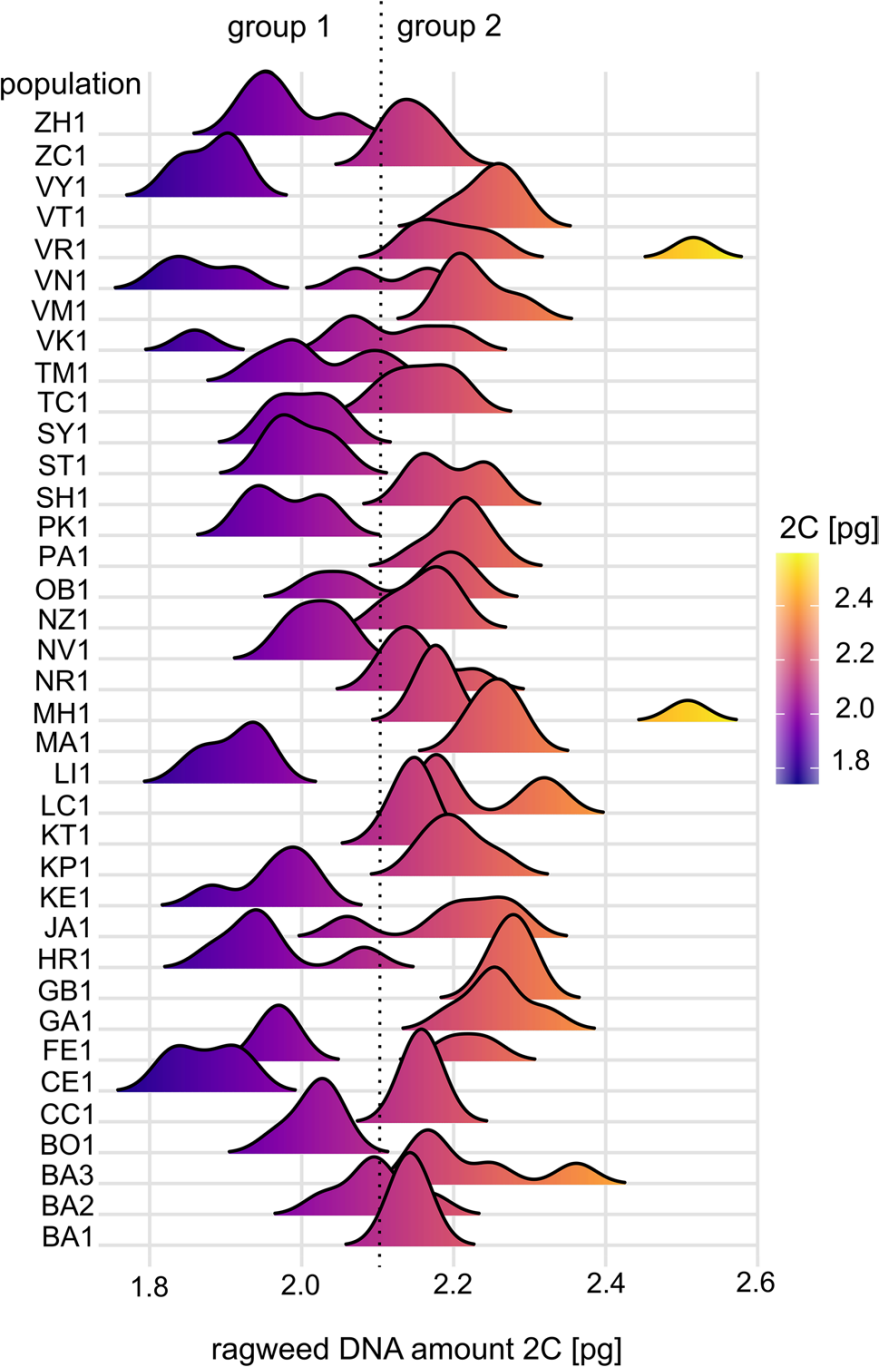
Interpopulation variability of *Ambrosia artemisiifolia* genome size in the study area. Density plots represent the variability in every population from Table 1. The populations with a smaller genome size (2C < 2.1 pg) belong to group 1, and the populations with a larger genome size (2C > 2.1 pg) belong to group 2. Some populations are mixed (e.g., FE1, OB1)

All data analyses were performed in the statistical software R (version 4.2.2). The individuals were experimentally divided into two groups: group 1 with a DNA amount (2C) smaller than 2.1 pg and group 2 with a DNA amount larger than 2.1 pg (Fig. 2). Applying the ctree() function of the “party” package (Hothorn et al. 2006), a non-parametric decision conditional inference tree was created to find the most important environmental factors affecting the ragweed genome size groups distribution in the study area. As environmental factors, geographical and meteorological variables such as altitude, latitude, longitude, anthropogenic habitats, and historical and current abovementioned climatic variables were tested.

Additionally, a linear mixed-effects model with individuals, populations, and measurement dates handled as random factors was conducted to test the impact of the abovementioned environmental factors on ragweed genome size distribution in the study area. The analysis was performed for all genome size measurements, but also without outliers (2C < 1.85 pg and 2C > 2.4 pg), using the “nlme” package (Pinheiro and Bates 2000) with the lme() function for the linear mixed-effects model. The effects of the previous factors on the DNA amount distribution in the studied area were also assessed using the cca() function of the “vegan” package (Oksanen 2012) for the constrained correspondence analysis (CCA). The differences in genome size in different anthropogenic habitats were assessed using the Kruskal–Wallis test, which was applied following Levene’s test for homogeneity using the Levene’s test() function of “cor” package (Guo 2020). Dunn’s test with Bonferroni correction was performed as a non-parametric post-hoc test using the Dunn’s test() function of “FSA” package (Ogle 2017).

The distribution of both genome size groups in the potential ragweed area (M. Hrabovský, unpublished data) was modelled using species distribution modelling (SDM). SDM is used to predict the distribution of species in an area based on environmental predictors (Farashi and Alizadeh-Noughani 2023). The ideal (presences) and unfavorable environmental conditions (pseudo-absences) are essential to determine for the modelling (Wang et al. 2023). For group 1 evaluation, environmental factors associated with group 2 were substituted for pseudo-absences, and vice versa. Models and predictions were calculated by the sdm() and ensemble() functions of the “sdm” package (Naimi and Araújo 2016) using the support vector machines (SVM) algorithm. The models were evaluated with cross-validation (tenfold), and the obtained true skill statistic (TSS) and area under the relative operating characteristic curve (AUC) values were higher than 0.7.

## Results

### DNA amount of ragweed

The mean value of the absolute DNA amount (2C) estimated from 185 individuals was 2.11 pg. The range of the DNA amount (2C) was spanning from 1.819 to 2.516 pg. The exact data for each population are depicted in Table 1 and Table S1. The 16 populations belong mostly to group 1 with a smaller genome size, and the 21 populations belong mostly to group 2 with a larger DNA amount, but some populations are mixed (Fig. 2). The analysis of herbarium specimens suggests that group 1 with a smaller genome was introduced to the territory of Slovakia first (Fig. S2).

**Table 1:** The absolute DNA amount of naturalized ragweed populations in the northern Pannonian Basin

*Pop*	*Hab*	*Group*	*Lat*	*Long*	*Alt* [m. a. s. l.]	2C* [pg] (min–max)	*CV [%]*
VT1	RH	2	47.75079	18.31760	105.3	2.247 (2.192–2.289)	1.5
VK1	RW	1	48.55485	22.10377	107.3	2.07 (1.859–2.205)	5.7
PA1	RS	2	47.73917	18.31495	108	2.208 (2.154–2.25)	1.4
MH1	AL	2	47.85146	18.68839	111	2.242 (2.159–2.508)	5.9
VM1	AL	2	47.8478	17.78612	112.4	2.228 (2.197–2.291)	1.6
NZ1	RS	2	47.96135	18.18768	115.3	2.162 (2.107–2.203)	1.5
JA1	RS	2	48.12879	18.02957	117.2	2.201 (2.06–2.285)	3.6
OB1	AL	2	47.77706	18.65024	117.4	2.133 (2.016–2.218)	3.7
NV1	AL	1	48.63933	21.685	119.1	2.02 (1.977–2.059)	1.5
GA1	RH	2	48.18709	17.71218	119.3	2.256 (2.197–2.321)	1.8
FE1	AL	1	48.76618	22.08028	119.6	2.069 (1.952–2.243)	6.0
ST1	RW	1	47.8021	18.68476	120.8	1.998 (1.96–2.047)	1.7
GB1	AL	2	47.86129	18.49871	121.5	2.277 (2.249–2.294)	0.7
BA3	RH	2	48.10522	17.10971	133	2.362 (2.151–2.361)	3.8
NR1	RH	2	48.28626	18.09509	136.4	2.155 (2.113–2.228)	1.9
SH1	RS	2	48.08334	18.94056	139.8	2.196 (2.151–2.244)	1.9
VN1	RS	1	48.66862	22.25716	142.8	1.966 (1.819–2.166)	6.7
KP1	AL	2	48.29594	17.44926	151.8	2.202 (2.157–2.26)	1.6
CC1	RS	2	48.23428	18.04753	152	2.158 (2.141–2.176)	0.5
VR1	AL	2	48.23695	18.33368	152.7	2.258 (2.142–2.516)	5.9
KT1	RS	2	48.6582	17.04409	157	2.146 (2.117–2.164)	0.7
BA1	RH	2	48.26141	16.96355	162	2.143 (2.126–2.16)	0.5
PK1	RS	1	48.29365	17.29239	167.5	1.979 (1.931–2.036)	2.1
BO1	RS	1	48.45007	17.50928	170.1	2.017 (1.969–2.048)	1.3
TM1	RS	1	48.34993	18.3644	170.6	2.024 (1.941–2.116)	3.2
BA2	RS	1	48.17538	16.99063	182.4	2.097 (2.029–2.17)	2.2
LC1	RS	2	48.34716	19.65759	187.1	2.234 (2.168–2.332)	3.1
MA1	RS	2	48.44702	17.07451	189.2	2.255 (2.22–2.283)	1.0
KE1	RS	1	48.68977	21.28078	195.9	1.965 (1.881–2.012)	2.4
ZC1	RW	2	48.48316	18.72734	217.8	2.145 (2.115–2.187)	1.3
HR1	RS	1	48.46828	19.95121	239.4	1.959 (1.884–2.082)	3.4
ZH1	RH	1	48.57629	18.83322	240.5	1.971 (1.923–2.052)	2.2
TC1	RS	2	48.35703	18.52149	263.5	2.159 (2.102–2.21)	1.8
SY1	RS	1	48.83974	19.11476	494.6	2.003 (1.956–2.051)	1.9
LI1	RS	1	49.38852	20.63251	775.3	1.911 (1.779–1.95)	2.0
VY1	RS	1	49.15063	20.25393	982	1.88 (1.835–1.91)	1.8
CE1	RS	1	48.90538	19.73179	1206.8	1.877 (1.824–1.926)	2.5

^*^Mean value of absolute DNA amount averaged per population; *Pop*, population code; *Hab*, habitat (*AL* arable land, *RH* ruderal habitats, *RS* roadsides, *RW* railways); *Group*—prevalent genome size group (1—2C < 2.1 pg, 2—2C > 2.1 pg); *Lat*, latitude; *Long*, longitude; *Alt*, altitude; *CV*, coefficient of variation of population

### DNA amount and environmental variables

**Fig. 3:**
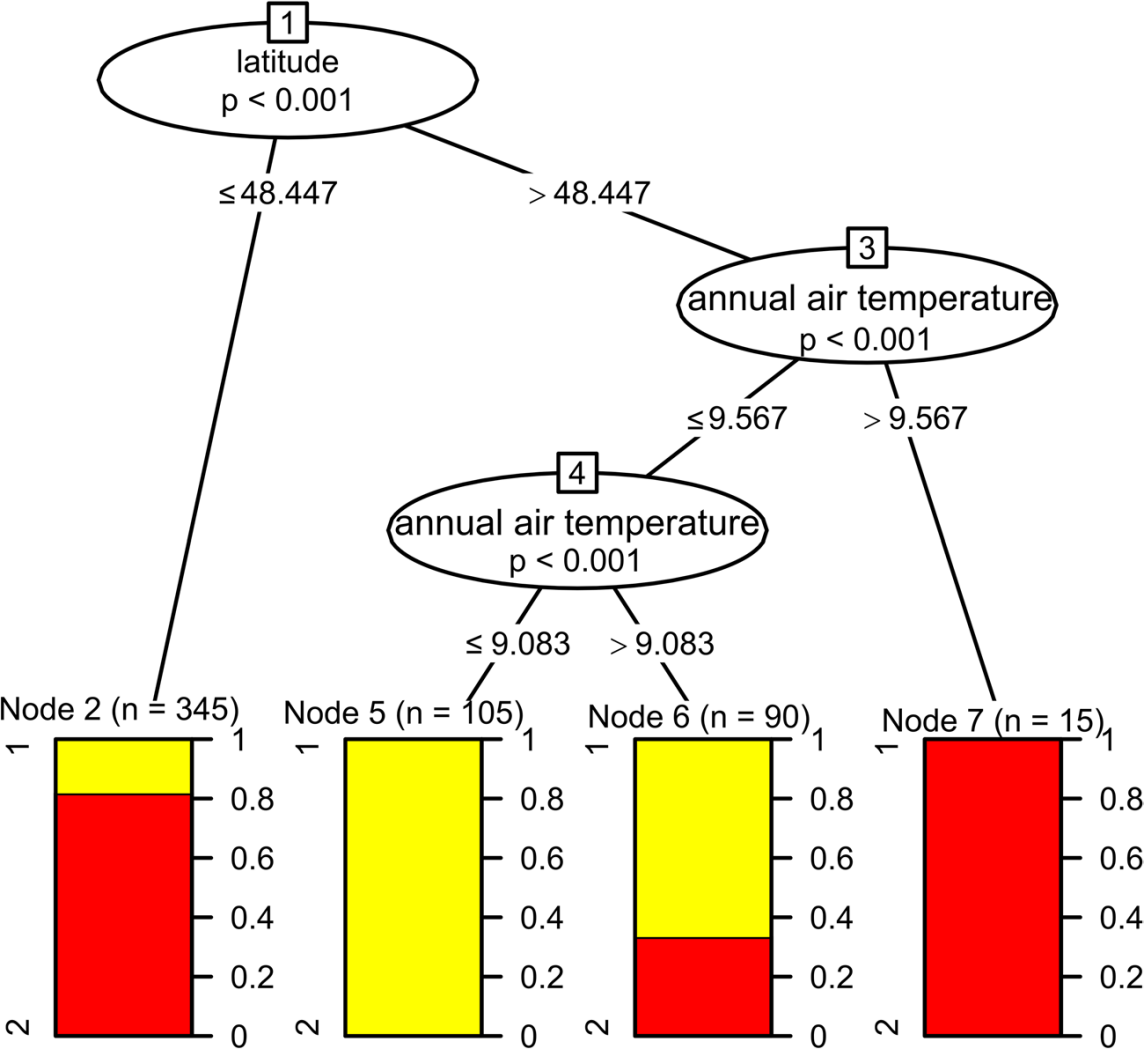
The conditional inference showing the main factors (latitude and mean annual air temperature in the historical period) affecting the distribution of ragweed genome size in the study area; yellow—group 1 (2C < 2.1 pg), red—group 2 (2C > 2.1 pg)

After dividing the populations into groups, 33% of Pannonian populations belong to group 1 and 67% to group 2. On the contrary, 86% of Carpathian populations are classified as group 1, whereas only 14% are in group 2. According to the conditional inference tree (Fig. 3), the main factor affecting the distribution of both groups in the studied area is latitude and mean annual air temperature in the historical period (1970 − 2000). Group 1 occurs mainly in areas with a latitude greater than 48.447 N (*p* < 0.001) and where the mean annual air temperature was below 9.567 °C (*p* < 0.001) during the time of ragweed introduction (Fig. S3). This redistribution of groups was also captured by SDM (Fig. S4).

**Fig. 4:**
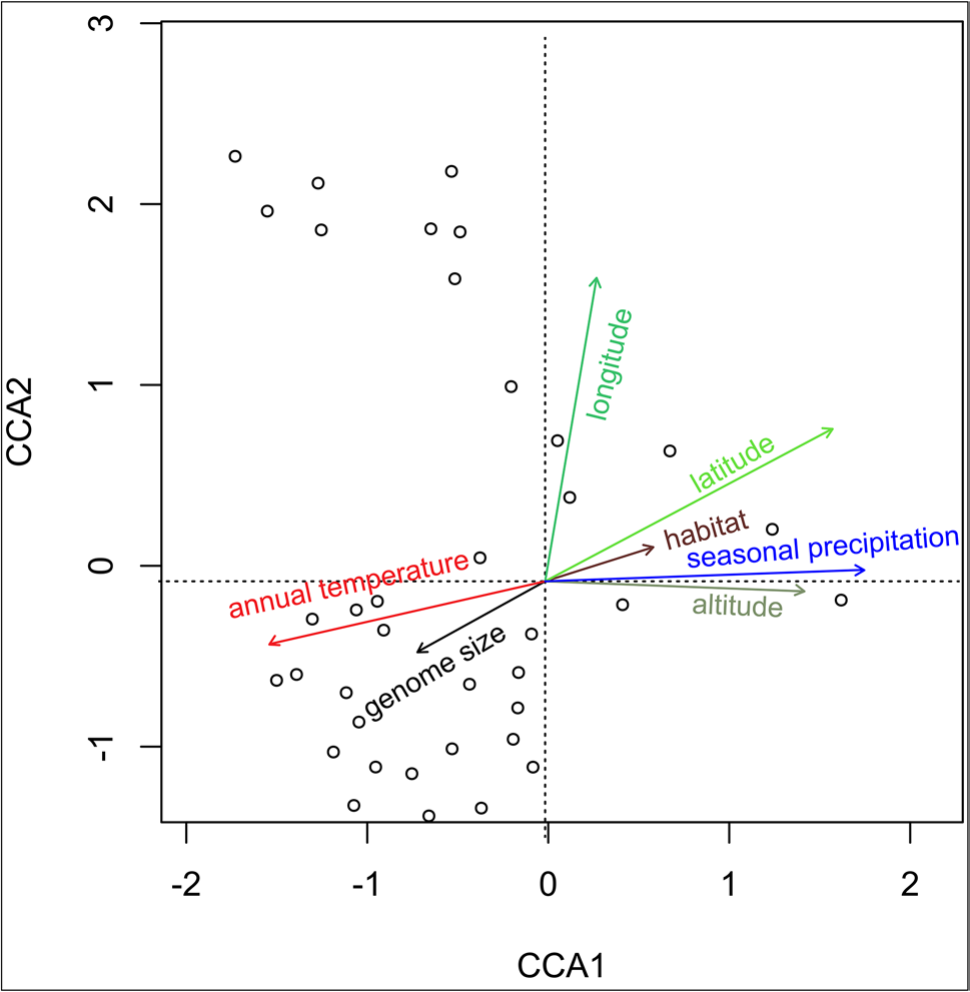
Results of the constrained correspondence analysis (CCA). CCA1 axis corresponds to the altitude and the seasonal May − October precipitation totals, CCA2 axis to longitude; points represent 37 studied ragweed populations. The acute angle between the arrows indicates a strong association between variables. The arrow length represents the strength of the correlation, which can be positive or negative according to the arrow direction. The permutation test (*n* = 999) confirmed the significance of the analysis (*F* = 1197, *p* < 0.001)

Results of the linear mixed-effects model (Table 2) indicated that there was a significant (*p* < 0.001) positive association between the estimated DNA amounts of group 1 and the current period mean annual air temperature (beta = 0.017, SE = 0.003, *R*^2^ marginal = 0.274), the mean May–October air temperature (beta = 0.015, SE = 0.003, *R*^2^ marginal = 0.265), and the mean December − March air temperature (beta = 0.021, SE = 0.004, *R*^2^ marginal = 0.290), and a significant negative association with the current period annual precipitation totals (beta =  − 0.0002, SE = 0.0004, *R*^2^ marginal = 0.173), and the seasonal May − October precipitation totals (beta =  − 0.0003, SE = 0.0001, *R*^2^ marginal = 0.193). Using historical meteorological data instead of the current period climate for model calculation results in stronger and statistically significant connections (higher marginal *R*^2^ and* t* values) between the DNA amount and the aforementioned environmental parameters. A significant negative association between geographical variables (altitude, latitude, and longitude) and the absolute DNA amount was also found by the linear mixed-effects models for group 1 (Table 2). The same regression trends (except longitude) were maintained for group 2, but with a low *R*^2^ (Table 2). In addition, a linear-mixed effects model was computed for the following scenarios: plants were not divided into two groups (Table S3), outliers were excluded (Table S4), and populations (Table S5) or measurement dates (Table S6) were used as random factors. No fundamental differences between the results of previous models were observed; therefore, a spatio-temporal bias resulting from different habitat selection or measurement dates can be excluded as the reason for the observed trends. CCA analysis yields similar results as the linear mixed-effects models. The first ordination axis CCA1 corresponds to the altitude and the seasonal May − October precipitation totals, while the second ordination axis CCA2 correlates to longitude (Fig. 4).

**Table 2:** Linear mixed-effects model coefficients and statistics for two different DNA amount groups of ragweed, and meteorological and geographical variables as fixed effects, and an individual plant (i.e., results from repeated measurements of the same plant) as a random effects

		Group 1 (2C < 2.1 pg)						Group 2 (2C > 2.1 pg)					
Variables (fixed effects)		DNA amount (intercept)	beta	SE	*t* (*p* value) df = 76	*R*^2^*c*	*R*^2^*m*	DNA amount (intercept)	beta	SE	*t* (*p* value) df = 108	*R*^2^*c*	*R*^2^*m*
*T*_mean_	■	1.808	0.0198	0.003	6.06***	0.991	0.323	1.897	0.0305	0.0135	2.25*	0.990	0.044
	□	1.810	0.017	0.003	5.38***	0.991	0.274	1.891	0.0279	0.012	2.3*	0.990	0.046
*T*_mean_ May–October	■	1.715	0.017	0.0029	5.78***	0.991	0.310	1.694	0.0305	0.0127	2.4*	0.990	0.050
	□	1.726	0.015	0.003	5.55***	0.991	0.265	1.693	0.028	0.0113	2.5*	0.990	0.054
*T*_mean_ December–March	■	1.977	0.024	0.004	6.46***	0.991	0.350	2.172	0.0217	0.0126	1.72 NS	0.990	0.026
	□	1.965	0.021	0.004	5.62***	0.991	0.290	2.161	0.0205	0.0112	1.82 NS	0.990	0.029
*P*	■	2.153	− 0.0003	0.00004	− 6.01***	0.991	0.318	2.284	− 0.0001	0.0001	− 1.49 NS	0.990	0.029
	□	2.109	− 0.0002	0.00004	− 4.01***	0.991	0.173	2.313	− 0.0001	0.0001	− 1.65 NS	0.990	0.024
*P* May–October	■	2.141	− 0.0008	0.00006	− 6.46***	0.991	0.350	2.254	− 0.0001	0.0001	− 1.2 NS	0.990	0.013
	□	2.111	− 0.0003	0.0001	− 4.23***	0.991	0.193	2.258	− 0.0001	0.0001	− 1.1 NS	0.990	0.011
*P* December–March	■	2.429	− 0.008	0.0002	− 3.73***	0.991	0.154	2.323	− 0.0008	0.0004	− 2.02*	0.990	0.036
	□	2.034	− 0.0004	0.0002	− 1.91NS	0.991	0.046	2.363	− 0.001	0.0004	− 2.69**	0.990	0.062
Altitude		2.018	− 0.0001	0.00002	− 5.97***	0.991	0.317	2.267	− 0.0004	0.0002	− 2.66**	0.990	0.059
Latitude		6.71	− 0.097	0.02	− 5.37***	0.991	0.271	5.40	− 0.066	0.024	− 2.77**	0.990	0.066
Longitude		2.283	− 0.015	0.006	− 3.25***	0.991	0.118	2.158	0.002	0.006	− 0.4 NS	0.990	0.001

^*^p < 0.05, **p < 0.01, ***p < 0.001

*NS*, non-significant; *T*_mean_, mean air temperature; *P*, total precipitation; black square, historical period (1970–2000), white square, current period (2020–2021); *SE*, standard error, *R*^2^*c*, conditional *R*^2^ for random and fixed effects, *R*^2^*m*, marginal *R*^2^ for fixed effects

### DNA amount and habitats

**Fig. 5:**
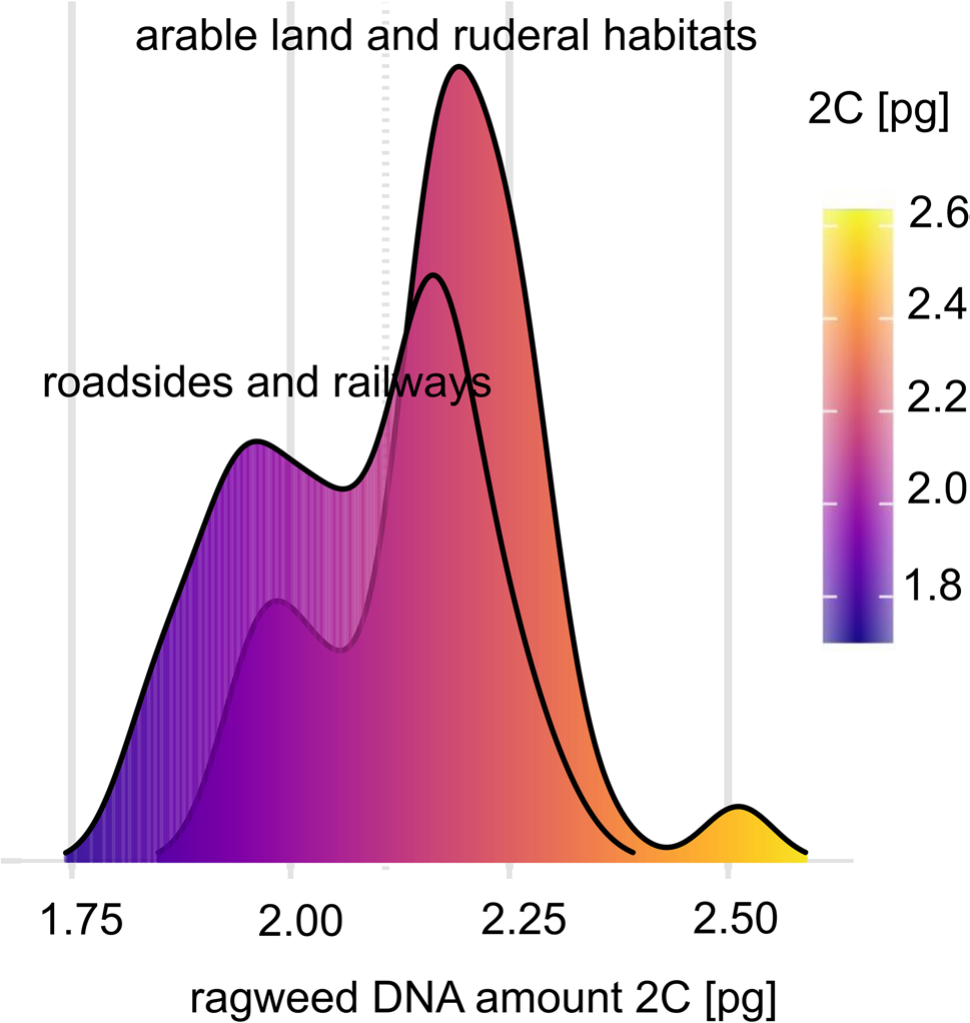
Differences in DNA amount at different biotope types. The smaller genome size (2C < 2.1 pg) is more frequent along roadsides and railways than in arable land and ruderal habitats

Based on Kruskal–Wallis test, the genome size seems to vary with selected types of anthropogenic biotopes such as roadsides, arable land, railways, or ruderal habitats (*χ*2 = 26.6, *p* < 0.001). A post-hoc test shows no differences between arable land and ruderal habitats (adjusted *p* = 1) and roadsides and railways (adjusted *p* = 1). However, these couples (Fig. 5) differ significantly from one another (adjusted *p* < 0.001).

## Discussion

### Intraspecific variation of *Ambrosia artemisiifolia* genome size

**Fig. 6:**
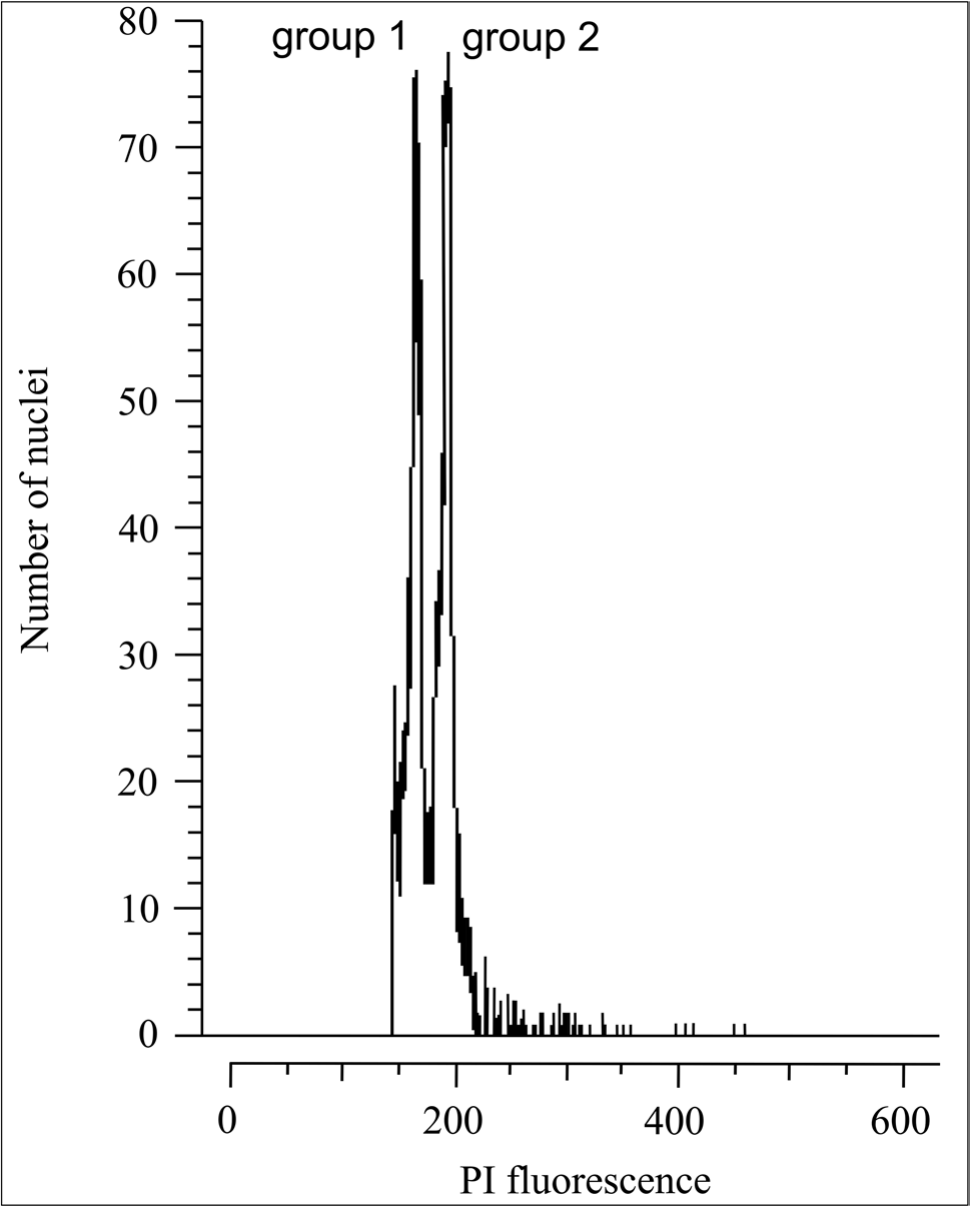
Flower cytometry histogram with the double peak showing the differences in the ragweed genome size between the selected samples of both genome size groups. The samples were co-chopped for the analysis. The ratio between peaks of group 1 (sample VN105) and group 2 (sample KP103) is 0.81

It is a controversial subject, whether there is any diversity in genome size below the level of a species (Greilhuber 1998). Despite the doubts, intraspecific variability of genome size was confirmed in Asteraceae family (Suda et al. 2007; Slovák et al. 2009; Dirkse et al. 2014) and in other flowering plants (e.g., Poaceae, Šmarda and Bureš 2006; Šmarda et al. 2008; Ranunculaceae, Cires et al. 2010; Amaryllidaceae, Duchoslav et al. 2013; Anacardiaceae, Aliyu 2014; Caprifoliaceae, Frajman et al. 2015; Caryophyllaceae, Terlević et al. 2022). Ragweed’s intraspecific variability has not yet been investigated. The known genome size values of the ragweed were determined by examining specimens collected from both its native North American area–2.08 pg/2C (Bai et al. 2012) and non-native Europe–2.12 pg/2C (Kubešová et al. 2010) or 2.18 to 2.27 pg/2C (Battlay et al. 2023). We estimated approximately the same genome size value averaged from 185 ragweed individuals (2.11 ± 0.3 pg/2C) as it was in North America or Europe. A noticeable disparity in the peak distance was observed between the sample with smaller genome size (2C < 2.1 pg) and the larger genome size sample (2C > 2.1 pg) (Fig. 6). Intraspecific genome size variability can be generally caused by a number of genomic mechanisms, such as transposable elements, tandem repeats, or recombination rate, often driven by environmental changes (Tiley and Burleigh 2015; Wang et al. 2021). Aneuploidy, as a possible source of variation, has not been observed in both native populations (Jones 1936; Bolkhovskikh et al. 1969; Bassett and Crompton 1975) and naturalized individuals within the study area (Májovský et al. 1974; Feráková and Javorčíková 1974). It is less likely that all plants in populations with smaller or larger genome sizes would be aneuploids. The observed variability can be clarified when the measured values are divided into two groups. It is likely that these groups represent different races that originated in North America. There are two genetic groups of ragweed in Europe (Gladieux et al. 2011), but it is not certain whether they correlate with the groups we have discovered. The first ragweed populations were brought to Slovakia from Canada (Hrabovský et al. 2016). We now estimated that their genome size was smaller. It might seem that the sizes of larger genomes are more difficult to estimate from herbarium specimens (Viruel et al. 2019) and therefore were not found. However, we also found sample with a larger genome from 1971. Such plants could have occurred earlier in the study area. The problem is the absence of a sufficient amount of ragweed herbarium collections that would help determine the exact period of introduction of the second ragweed group with a larger genome size to Slovakia. Ragweed was brought to most European countries from the USA (Makra et al. 2015). Therefore, only the group with a larger genome size has been detected in Europe so far (Kubešová et al. 2010; Battlay et al. 2023). Most likely, this group migrated into Slovakia from Hungary and mixed with already imported populations with smaller genome size.

### The importance of climatic factors for ragweed genome size selection

Latitude is the most evident factor that correlates with the genome size of many plant species. In the northern hemisphere, the genome size increases from the equator to the temperate zone and decreases again towards the pole (Yu et al. 2018; Bureš et al. 2024). Monoploid genome size in *Ambrosia* species increases from subtropical regions to the temperate zone (Sliwinska et al. 2009; Zonneveld 2019), but in temperate *Ambrosia artemisiifolia*, it is again smaller (Kubešová et al. 2010; Pustahija et al. 2013; Zonneveld 2019). We observed two ragweed genome size groups that correlate with latitude. This might be related to the shorter life cycle of ragweed, which grows in northern latitudes (Scalone et al. 2016). It can be assumed that the different ecological preferences of the groups, which are dispersed throughout the region, are the reason for the observed correlations between ragweed genome size and other environmental factors. However, our analyses showed that there are associations between genome size and environmental factors within each group. These associations have comparable regression trends but different *R*^2^ values. Plants of group 1 growing in more northern latitudes seem to be under higher selection pressure than plants of group 2. A similar case was observed in perennial *Phragmites australis*, where different genome size groups are known, but their monoploid genome size had opposing correlation trends (Meyerson et al. 2024). Relationships between variability in the genome size and various environmental factors due to natural selection are also known in annual plants such as *Eragrostis* (Hutang et al. 2023) or wild *Zea mays* (Rayburn and Auger 1990; Bilinski et al. 2018). The negative correlation between genome size, latitude, and seasonal precipitation and a positive association with the annual mean temperature, as we observed in ragweed, are known in sunflowers (Qiu et al. 2019). This may be explained by their genetic relatedness (Urbatsch et al. 2000).

The phenotypic variability of the ragweed is contingent upon factors such as temperature, latitude, and longitude (Dickerson and Sweet 1971; Leiblein-Wild and Tackenberg 2014). Our investigation has augmented the current understanding of this subject matter by highlighting the correlation between genome size and the aforementioned factors. In the studied area, the latitude, longitude, and elevation are in a close relationship with temperature-precipitation regime (Čimo et al. 2020). This regime exhibits a marked shift from west to east, owing to the alternating maritime transition zone and continentality (Vilček et al. 2016). The influence of continentality on ragweed genome size distribution in studied area, manifested by increasing temperature and decreasing precipitation, is reflected in our results. Thus, indications of selection of group 1 with smaller genome can be observed in areas with unfavorable climatic conditions due to continentality or elevation, while in areas characterized by optimal conditions, genetic drift could account for the observed variability, and hence the selection is less evident. Furthermore, the average genome size values are significantly lower in regions with unfavorable climatic conditions for ragweed survival. This is in line with the large genome constraint hypothesis (Knight et al. 2005), according to which extreme environmental conditions constrain species with large genomes. Genome size reduction helps invasive plants adapt better to a new environment (Lavergne et al. 2010). The reason for the selection of a smaller genome in mountain regions could be attributed to an adaptive strategy to cope with a shorter vegetation period, given that plants with reduced genome sizes have comparatively shorter life cycle (Bennett 1972; Leitch and Bennett 2007). Ragweed populations that have evolved to a shorter life cycle as a result of environmental conditions are known (Hodgins and Rieseberg 2011; Scalone et al. 2016; Gentili et al. 2018). Even outside mountainous areas, the temperature and precipitation can limit the life cycle of the ragweed (Dickerson 1968; Bassett and Crompton 1975). Although ragweed thrives in warm environments, its geographic range is restricted by low winter temperatures and limited precipitation during the growing season (Shrestha et al. 1999; Gentili et al. 2019). The positive correlation between the ragweed DNA amount with winter and summer temperatures indicates the influences of warm summer months as well as a cold winter period for the adaptive DNA amount variation. The precipitation regime, on the other hand, appears to be an effective selective factor for genome size only during the growing season (May–October).

### Climatic change and adaptation reflected in genome size

The results of our study do not provide definitive evidence as to whether the emergence of genome size variability occurred during the naturalization period (1970 − 2000) or whether it is an ongoing selective process that extends to the present. The observed differences in meteorological variables (i.e., an increase in mean temperature of 0.78–1.40 °C and an increase in precipitation of 42.6–80.9 mm) between historical and current periods at the study sites did not have a significant impact on the statistical results. This is because the correlation between historical and current period mean temperature and precipitation is strong (*ρ* = 0.99, *p* < 0.001). This fails to provide definitive evidence as to whether the genome size variation is more strongly associated with historical environmental factors or the current climate. However, climate change often produces new selection pressure (Hoffmann and Sgrò 2011), and the evolutionary response to such change can be rapid (Whitney and Gabler 2008).

### Stressful conditions and ragweed genome size

Anthropogenic biotopes such as roadsides, arable land, railways, or ruderal habitats are often exposed to mixtures of various factors (e.g., traffic emissions, alkalinization of soils, pesticides) with a stressful effect on the plant (Klumpp et al. 2006). We found that the genome size of ragweed growing along the road and rail network is lower than that in arable land and ruderal habitats. The observed difference between ragweed genome size in different anthropogenic habitats can be caused by the aforementioned relationships between genome size and environmental factors, since ragweed tends to grow primarily along roads and railways in the mountains, which are associated with colder climates, while it grows in every anthropogenic habitat in warmer and drier lowland areas (Hrabovský and Mičieta 2018). However, along roads and railways, unlike ruderal habitats and fields, there is frequent mowing, and only individuals with a shorter life cycle can produce seeds under disturbation pressure.

In conclusion, climate could be one of the factors in shaping the distribution of two *Ambrosia artemisiifolia* genome size groups in the study area. It is worth noting that due to the localized nature of our research, the identified relationships may lack generalizability to other European or non-European regions. In the future, it will be interesting to monitor the genome size of more ragweed populations in mountain regions where naturalization processes are currently underway.

### Supplementary Information

Below is the link to the electronic supplementary material.

Supplementary file1 (PDF 896 KB)

## Data Availability

All data generated or analyzed during this study are included in this published article.
