# Supplementary material for: Environmental impacts on intraspecific variation in Ambrosia artemisiifolia genome size in Slovakia, Central Europe
Source: Environ Sci Pollut Res Int. 2024 May 2;31(23):33960–74. doi: 10.1007/s11356-024-33410-x (PMC11136817; doi:10.1007/s11356-024-33410-x)
Supplement: Supplementary file 1 — Supplementary file1 (PDF 896 KB) [file 11356_2024_33410_MOESM1_ESM.pdf]

Estimated ragweed DNA amounts

Supplementary Table 1. Flow cytometry measurements data for ragweed genome size

Sample	DNA amount 2C [pg]	measurment 1. [pg]	measurment date 1.	measurment 2. [pg]	measurment date 2.	measurment 3. [pg]	measurment date 3.
BA101	2.159788633	2.160868527	16.1.2023	2.146438633	17.1.2023	2.172058739	18.1.2023
BA102	2.139166939	2.140450439	16.1.2023	2.125816939	17.1.2023	2.151233439	18.1.2023
BA103	2.125874296	2.127149821	16.1.2023	2.127149821	17.1.2023	2.123323247	18.1.2023
BA104	2.146379954	2.148097058	16.1.2023	2.149600726	17.1.2023	2.141442078	18.1.2023
BA105	2.141559759	2.143058851	16.1.2023	2.14413038	17.1.2023	2.137490046	18.1.2023
BA201	2.097147437	2.09861544	6.9.2022	2.098407437	7.9.2022	2.094419434	8.9.2022
BA202	2.107060965	2.108535908	6.9.2022	2.115586022	7.9.2022	2.097060965	8.9.2022
BA203	2.082954587	2.084412655	6.9.2022	2.085454862	7.9.2022	2.078996244	8.9.2022
BA204	2.169611467	2.171347156	6.9.2022	2.17243283	7.9.2022	2.165054415	8.9.2022
BA205	2.028962731	2.030180109	6.9.2022	2.031804253	7.9.2022	2.024903832	8.9.2022
BA301	2.250087742	2.251212786	16.1.2023	2.236737742	17.1.2023	2.262312698	18.1.2023
BA302	2.151252587	2.150176961	16.1.2023	2.152512587	17.1.2023	2.151068213	18.1.2023
BA303	2.183368165	2.184678186	16.1.2023	2.182930443	17.1.2023	2.182495866	18.1.2023
BA304	2.164389388	2.162874315	16.1.2023	2.164388327	17.1.2023	2.165905521	18.1.2023
BA305	2.361313197	2.359896409	16.1.2023	2.347963197	17.1.2023	2.376079985	18.1.2023
BO101	2.019759096	2.021172927	15.12.2022	2.022789866	16.12.2022	2.015314495	19.12.2022
BO102	2.047598162	2.048826721	15.12.2022	2.034248162	16.12.2022	2.059719603	19.12.2022
BO103	2.033254679	2.034677957	15.12.2022	2.019904679	16.12.2022	2.045181401	19.12.2022
BO104	2.017196208	2.015784171	15.12.2022	2.018456208	16.12.2022	2.017348245	19.12.2022
BO105	1.968817911	1.969999202	15.12.2022	1.971378201	16.12.2022	1.96507633	19.12.2022
CC101	2.17632828	2.17480485	23.1.2023	2.17480485	24.1.2023	2.17937514	25.1.2023
CC102	2.155799278	2.157092758	23.1.2023	2.157059278	24.1.2023	2.153245798	25.1.2023
CC103	2.157788374	2.159514605	23.1.2023	2.159048374	24.1.2023	2.154802143	25.1.2023
CC104	2.141488371	2.140203478	23.1.2023	2.142748371	24.1.2023	2.141513264	25.1.2023
CC105	2.157851076	2.159145787	23.1.2023	2.160657189	24.1.2023	2.153750253	25.1.2023
CE101	1.926380616	1.92792172	10.10.2022	1.913030616	11.10.2022	1.938189512	12.10.2022
CE102	1.824212707	1.825672077	10.10.2022	1.825472707	11.10.2022	1.821493337	12.10.2022
CE103	1.836585334	1.837870944	10.10.2022	1.838789879	11.10.2022	1.833095179	12.10.2022
CE104	1.910258707	1.911786914	10.10.2022	1.896908707	11.10.2022	1.9220805	12.10.2022
CE105	1.875765322	1.874827439	10.10.2022	1.862415322	11.10.2022	1.890053205	12.10.2022
FE101	2.242765462	2.243886845	6.9.2022	2.242540513	7.9.2022	2.241869029	8.9.2022
FE102	2.194405646	2.192650121	6.9.2022	2.193746447	7.9.2022	2.19682037	8.9.2022
FE103	1.975668348	1.977051316	6.9.2022	1.962318348	7.9.2022	1.98763538	8.9.2022
FE104	1.952330939	1.953697571	6.9.2022	1.954674419	7.9.2022	1.948620827	8.9.2022
FE105	1.978461972	1.977274895	6.9.2022	1.978263532	7.9.2022	1.979847489	8.9.2022
GA101	2.257595568	2.259175885	24.10.2022	2.260983226	25.10.2022	2.252627593	26.10.2022
GA102	2.258033618	2.259840045	24.10.2022	2.258484141	25.10.2022	2.255776668	26.10.2022
GA103	2.248500151	2.250298951	24.10.2022	2.250298951	25.10.2022	2.244902551	26.10.2022
GA104	2.196640539	2.198178187	24.10.2022	2.198178187	25.10.2022	2.193565242	26.10.2022
GA105	2.321054004	2.322678742	24.10.2022	2.324536885	25.10.2022	2.315946385	26.10.2022
GB101	2.248532172	2.250106145	24.10.2022	2.250106145	25.10.2022	2.245384227	26.10.2022

GB102	2.294465201	2.296071327	24.10.2022	2.281115201	25.10.2022	2.306209075	26.10.2022
GB103	2.2932844	2.294660371	24.10.2022	2.296496099	25.10.2022	2.28869673	26.10.2022
GB104	2.272249301	2.2740671	24.10.2022	2.258899301	25.10.2022	2.283781502	26.10.2022
GB105	2.274312825	2.275677413	24.10.2022	2.275677413	25.10.2022	2.27158365	26.10.2022
HR101	2.082027017	2.080361395	23.1.2023	2.068677017	24.1.2023	2.097042639	25.1.2023
HR102	1.942349746	1.943903626	23.1.2023	1.942542893	24.1.2023	1.940602719	25.1.2023
HR103	1.929432016	1.930589675	23.1.2023	1.931748029	24.1.2023	1.925958344	25.1.2023
HR104	1.884311139	1.88280369	23.1.2023	1.885571139	24.1.2023	1.884558588	25.1.2023
HR105	1.955330475	1.95435281	23.1.2023	1.956590475	24.1.2023	1.95504814	25.1.2023
JA101	2.185981198	2.187511385	17.10.2022	2.189042643	18.10.2022	2.181389566	19.10.2022
JA102	2.284531941	2.28590266	17.10.2022	2.287045611	18.10.2022	2.280647551	19.10.2022
JA103	2.257162772	2.256034191	17.10.2022	2.254906174	18.10.2022	2.260547952	19.10.2022
JA104	2.059884584	2.061120515	17.10.2022	2.05967773	18.10.2022	2.058855507	19.10.2022
JA105	2.219665574	2.220775407	17.10.2022	2.220925574	18.10.2022	2.217295741	19.10.2022
KE101	2.012239712	2.011233592	16.1.2023	2.011233592	17.1.2023	2.014251952	18.1.2023
KE102	1.999143318	1.997943832	16.1.2023	1.99694486	17.1.2023	2.002541262	18.1.2023
KE103	1.97622389	1.977212002	16.1.2023	1.975827954	17.1.2023	1.975631715	18.1.2023
KE104	1.880905644	1.882034187	16.1.2023	1.867555644	17.1.2023	1.893127101	18.1.2023
KE105	1.954864247	1.953886815	16.1.2023	1.955449924	17.1.2023	1.955256002	18.1.2023
KP101	2.179162388	2.180905718	12.9.2022	2.179815265	13.9.2022	2.176766181	14.9.2022
KP102	2.20188696	2.20364847	12.9.2022	2.20314696	13.9.2022	2.19886545	14.9.2022
KP103	2.259861702	2.258279799	12.9.2022	2.259634767	13.9.2022	2.261670541	14.9.2022
KP104	2.156826863	2.158336642	12.9.2022	2.15941581	13.9.2022	2.152728137	14.9.2022
KP105	2.211696886	2.213245074	12.9.2022	2.21501567	13.9.2022	2.206829914	14.9.2022
KT101	2.148733744	2.147444504	24.10.2022	2.14873297	25.10.2022	2.150023758	26.10.2022
KT102	2.117261904	2.115779821	24.10.2022	2.115779821	25.10.2022	2.120226071	26.10.2022
KT103	2.145621579	2.14669439	24.10.2022	2.148411745	25.10.2022	2.141758602	26.10.2022
KT104	2.164467827	2.165766508	24.10.2022	2.167499121	25.10.2022	2.160137852	26.10.2022
KT105	2.152022838	2.15030122	24.10.2022	2.1515914	25.10.2022	2.154175894	26.10.2022
LC101	2.167787016	2.169087688	12.9.2022	2.170172232	13.9.2022	2.164101128	14.9.2022
LC102	2.182613864	2.184359955	12.9.2022	2.183873864	13.9.2022	2.179607773	14.9.2022
LC103	2.180640256	2.181730576	12.9.2022	2.167290256	13.9.2022	2.192899936	14.9.2022
LC104	2.305899126	2.307052076	12.9.2022	2.308205602	13.9.2022	2.302439701	14.9.2022
LC105	2.332415356	2.331015907	12.9.2022	2.33288072	13.9.2022	2.333349442	14.9.2022
LI101	1.856832637	1.85813242	16.1.2023	1.856831727	17.1.2023	1.855533764	18.1.2023
LI102	1.951280261	1.952841285	16.1.2023	1.952540261	17.1.2023	1.948459237	18.1.2023
LI103	1.934837962	1.933677059	16.1.2023	1.935030633	17.1.2023	1.935806194	18.1.2023
LI104	1.884555117	1.885874306	16.1.2023	1.887194418	17.1.2023	1.880596628	18.1.2023
LI105	1.931970119	1.93061774	16.1.2023	1.918620119	17.1.2023	1.946672498	18.1.2023
MA101	2.283464484	2.284606216	15.12.2022	2.286205441	16.12.2022	2.279581795	19.12.2022
MA102	2.241247132	2.242367756	15.12.2022	2.243937413	16.12.2022	2.237436227	19.12.2022
MA103	2.274221258	2.272856725	15.12.2022	2.260871258	16.12.2022	2.288935791	19.12.2022
MA104	2.220201339	2.22131144	15.12.2022	2.206851339	16.12.2022	2.232441238	19.12.2022
MA105	2.254783553	2.25658738	15.12.2022	2.25839265	16.12.2022	2.249370629	19.12.2022
MH101	2.508029659	2.506023235	6.9.2022	2.494679659	7.9.2022	2.523386083	8.9.2022
MH102	2.178090319	2.176347847	6.9.2022	2.164740319	7.9.2022	2.193182791	8.9.2022
MH103	2.179531119	2.178441353	6.9.2022	2.179530574	7.9.2022	2.180621429	8.9.2022
MH104	2.159413144	2.157685613	6.9.2022	2.146063144	7.9.2022	2.174490675	8.9.2022

MH105	2.185697822	2.187009241	6.9.2022	2.172347822	7.9.2022	2.197736403	8.9.2022
NR101	2.15398481	2.155492599	6.9.2022	2.14063481	7.9.2022	2.165827021	8.9.2022
NR102	2.123195693	2.121709456	6.9.2022	2.124455693	7.9.2022	2.12342193	8.9.2022
NR103	2.228269883	2.230052499	6.9.2022	2.214919883	7.9.2022	2.239837267	8.9.2022
NR104	2.112638125	2.113694444	6.9.2022	2.099288125	7.9.2022	2.124931806	8.9.2022
NR105	2.154522128	2.155599389	6.9.2022	2.157323869	7.9.2022	2.150643126	8.9.2022
NV101	1.99762689	1.998625703	17.10.2022	1.99888689	18.10.2022	1.995368077	19.10.2022
NV102	1.976878759	1.977867198	17.10.2022	1.963528759	18.10.2022	1.98924032	19.10.2022
NV103	2.059293525	2.06094096	17.10.2022	2.060553525	18.10.2022	2.05638609	19.10.2022
NV104	2.04591139	2.047548119	17.10.2022	2.047548119	18.10.2022	2.042637932	19.10.2022
NV105	2.019927641	2.021139598	17.10.2022	2.022756509	18.10.2022	2.015886816	19.10.2022
NZ101	2.20306381	2.201301359	23.1.2023	2.20432381	24.1.2023	2.203566261	25.1.2023
NZ102	2.107443679	2.108708145	23.1.2023	2.10997337	24.1.2023	2.103649522	25.1.2023
NZ103	2.178535631	2.179842752	23.1.2023	2.165185631	24.1.2023	2.19057851	25.1.2023
NZ104	2.176565923	2.178307176	23.1.2023	2.176782361	24.1.2023	2.174608233	25.1.2023
NZ105	2.141980612	2.143479998	23.1.2023	2.143479998	24.1.2023	2.138981839	25.1.2023
OB101	2.196667179	2.198424513	8.2.2023	2.198424513	9.2.2023	2.193152512	10.2.2023
OB102	2.166798982	2.168315741	8.2.2023	2.170050394	9.2.2023	2.162030811	10.2.2023
OB103	2.21782747	2.219158166	8.2.2023	2.220711577	9.2.2023	2.213612666	10.2.2023
OB104	2.016057893	2.014646652	8.2.2023	2.014646652	9.2.2023	2.018880374	10.2.2023
OB105	2.065499284	2.066738584	8.2.2023	2.052149284	9.2.2023	2.077609984	10.2.2023
PA101	2.1535864	2.154663193	12.9.2022	2.1548464	13.9.2022	2.151249607	14.9.2022
PA102	2.213250898	2.214800174	12.9.2022	2.215907574	13.9.2022	2.209044947	14.9.2022
PA103	2.212274121	2.213380258	12.9.2022	2.214486948	13.9.2022	2.208955157	14.9.2022
PA104	2.250484296	2.249134005	12.9.2022	2.250933313	13.9.2022	2.25138557	14.9.2022
PA105	2.208280674	2.210047299	12.9.2022	2.211594332	13.9.2022	2.203200392	14.9.2022
PK101	1.970399648	1.971975968	24.10.2022	1.957049648	25.10.2022	1.982173328	26.10.2022
PK102	2.019679953	2.021295697	24.10.2022	2.021295697	25.10.2022	2.016448465	26.10.2022
PK103	1.93103012	1.931995635	24.10.2022	1.930836438	25.10.2022	1.930258287	26.10.2022
PK104	1.937877286	1.93671456	24.10.2022	1.924527286	25.10.2022	1.952390012	26.10.2022
PK105	2.036114305	2.037539585	24.10.2022	2.038558355	25.10.2022	2.032244975	26.10.2022
SH101	2.242152412	2.243721919	24.10.2022	2.241926941	25.10.2022	2.240808376	26.10.2022
SH102	2.186251226	2.187344352	24.10.2022	2.189094227	25.10.2022	2.182315099	26.10.2022
SH103	2.243784469	2.242438198	24.10.2022	2.243559417	25.10.2022	2.245355791	26.10.2022
SH104	2.151018272	2.152093781	24.10.2022	2.137668272	25.10.2022	2.163292763	26.10.2022
SH105	2.154300979	2.15580899	24.10.2022	2.157102475	25.10.2022	2.149991472	26.10.2022
ST101	1.959745952	1.95837413	17.10.2022	1.961005952	18.10.2022	1.959857774	19.10.2022
ST102	2.025593614	2.026606411	17.10.2022	2.027822375	18.10.2022	2.022352057	19.10.2022
ST103	1.971449492	1.973026652	17.10.2022	1.973026652	18.10.2022	1.968295173	19.10.2022
ST104	2.046872701	2.047896137	17.10.2022	2.033522701	18.10.2022	2.059199265	19.10.2022
ST105	1.98633039	1.987720821	17.10.2022	1.989310998	18.10.2022	1.981959351	19.10.2022
SY101	1.970938581	1.97192405	10.10.2022	1.973304397	11.10.2022	1.967587296	12.10.2022
SY102	1.956917677	1.955352143	10.10.2022	1.958177677	11.10.2022	1.957223211	12.10.2022
SY103	2.031228471	2.032447208	10.10.2022	2.034073166	11.10.2022	2.027165039	12.10.2022
SY104	2.051458633	2.052689508	10.10.2022	2.053921122	11.10.2022	2.047765269	12.10.2022
SY105	2.004388454	2.00338626	10.10.2022	2.00338626	11.10.2022	2.006392842	12.10.2022
TC101	2.190783497	2.189688105	15.12.2022	2.188374292	16.12.2022	2.194288093	19.12.2022
TC102	2.131771695	2.133050758	15.12.2022	2.133031695	16.12.2022	2.129232632	19.12.2022

TC103	2.21023374	2.21155988	15.12.2022	2.21266566	16.12.2022	2.20647568	19.12.2022
TC104	2.102029569	2.100978554	15.12.2022	2.088679569	16.12.2022	2.116430584	19.12.2022
TC105	2.158704625	2.159783977	15.12.2022	2.161295826	16.12.2022	2.155034072	19.12.2022
TM101	2.001664561	2.000063229	17.10.2022	2.001263267	18.10.2022	2.003667186	19.10.2022
TM102	1.940603691	1.941768053	17.10.2022	1.941768053	18.10.2022	1.938274967	19.10.2022
TM103	2.077017624	2.078056133	17.10.2022	2.063667624	18.10.2022	2.089329115	19.10.2022
TM104	2.115749965	2.117019415	17.10.2022	2.102399965	18.10.2022	2.127830515	19.10.2022
TM105	1.983427002	1.984617058	17.10.2022	1.985807828	18.10.2022	1.979856119	19.10.2022
VK101	2.205340155	2.207104427	8.2.2023	2.191990155	9.2.2023	2.216925883	10.2.2023
VK102	2.052005176	2.053236379	8.2.2023	2.053265176	9.2.2023	2.049513973	10.2.2023
VK103	2.152664459	2.151157594	8.2.2023	2.153924459	9.2.2023	2.152911324	10.2.2023
VK104	1.85931838	1.858202789	8.2.2023	1.857087867	9.2.2023	1.862664484	10.2.2023
VK105	2.081940192	2.08339755	8.2.2023	2.082355851	9.2.2023	2.080067175	10.2.2023
VM101	2.291045486	2.292649218	23.1.2023	2.292305486	24.1.2023	2.288181754	25.1.2023
VM102	2.2120343	2.210928283	23.1.2023	2.1986843	24.1.2023	2.226490317	25.1.2023
VM103	2.242101995	2.243223046	23.1.2023	2.243361995	24.1.2023	2.239720944	25.1.2023
VM104	2.198845721	2.200165028	23.1.2023	2.200165028	24.1.2023	2.196207106	25.1.2023
VM105	2.197488359	2.19924635	23.1.2023	2.198748359	24.1.2023	2.194470368	25.1.2023
VN101	1.855267588	1.856751802	8.2.2023	1.858051528	9.2.2023	1.850999434	10.2.2023
VN102	1.81946772	1.820377454	8.2.2023	1.80611772	9.2.2023	1.831907986	10.2.2023
VN103	2.071461037	2.069803868	8.2.2023	2.068355005	9.2.2023	2.076224237	10.2.2023
VN104	2.165741881	2.166824752	8.2.2023	2.16574134	9.2.2023	2.164659551	10.2.2023
VN105	1.916703392	1.91574504	8.2.2023	1.903353392	9.2.2023	1.931011744	10.2.2023
VR101	2.169746086	2.171481883	10.10.2022	2.156396086	11.10.2022	2.181360289	12.10.2022
VR102	2.208922594	2.210027055	10.10.2022	2.211353072	11.10.2022	2.205387655	12.10.2022
VR103	2.141510874	2.140011816	10.10.2022	2.138299807	11.10.2022	2.146220999	12.10.2022
VR104	2.252697926	2.250895768	10.10.2022	2.239347926	11.10.2022	2.267850084	12.10.2022
VR105	2.515545419	2.514036092	10.10.2022	2.515544513	11.10.2022	2.517055652	12.10.2022
VT101	2.289014777	2.290159284	10.10.2022	2.290159284	11.10.2022	2.286725762	12.10.2022
VT102	2.235289296	2.236853999	10.10.2022	2.236853999	11.10.2022	2.232159891	12.10.2022
VT103	2.267654195	2.268788022	10.10.2022	2.254304195	11.10.2022	2.279870368	12.10.2022
VT104	2.253566836	2.252214696	10.10.2022	2.253791246	11.10.2022	2.254694566	12.10.2022
VT105	2.191968106	2.193283287	10.10.2022	2.178618106	11.10.2022	2.204002925	12.10.2022
VY101	1.905962752	1.90710633	15.12.2022	1.906152776	16.12.2022	1.90462915	19.12.2022
VY102	1.909159414	1.91030491	15.12.2022	1.910419414	16.12.2022	1.906753918	19.12.2022
VY103	1.835260108	1.836361264	15.12.2022	1.821910108	16.12.2022	1.847508952	19.12.2022
VY104	1.849551068	1.848256382	15.12.2022	1.836201068	16.12.2022	1.864195754	19.12.2022
VY105	1.902013934	1.902964941	15.12.2022	1.904297016	16.12.2022	1.898779845	19.12.2022
ZC101	2.115283266	2.114225624	8.2.2023	2.101933266	9.2.2023	2.129690908	10.2.2023
ZC102	2.186990011	2.188520904	8.2.2023	2.173640011	9.2.2023	2.198809118	10.2.2023
ZC103	2.159871088	2.161167011	8.2.2023	2.162247594	9.2.2023	2.156198659	10.2.2023
ZC104	2.11758618	2.116527387	8.2.2023	2.118220609	9.2.2023	2.118010544	10.2.2023
ZC105	2.144932636	2.146219596	8.2.2023	2.146219596	9.2.2023	2.142358717	10.2.2023
ZH101	2.052115799	2.053347068	12.9.2022	2.051909726	13.9.2022	2.051090603	14.9.2022
ZH102	1.923336943	1.924490945	12.9.2022	1.924490945	13.9.2022	1.921028939	14.9.2022
ZH103	1.961899103	1.960329584	12.9.2022	1.960329584	13.9.2022	1.965038142	14.9.2022
ZH104	1.974637218	1.975624537	12.9.2022	1.961287218	13.9.2022	1.986999899	14.9.2022
ZH105	1.942353349	1.943324526	12.9.2022	1.941769866	13.9.2022	1.941965655	14.9.2022

Supplementary Table 2. Intrapopulation variability of common ragweed genome size estimated from 25 seedlings per population.

Population	DNA amount 2C [pg]				
P1, Kentucky, USA	2.2612	2.1923	2.3464	2.3901	2.2406
	2.2979	2.2688	2.1285	2.2304	2.2292
	2.2817	2.3131	2.2969	2.2788	2.2241
	2.2325	2.2917	2.3035	2.2584	2.2259
	2.2816	2.1815	2.1892	2.3002	2.2732
P2, Croatia	2.1849	2.293	2.1491	2.1922	2.2064
	2.2861	2.3264	2.1704	2.1559	2.1055
	2.1715	2.1904	2.1579	2.1554	2.1946
	2.2154	2.2213	2.2351	2.2414	2.1982
	2.2161	2.2264	2.1704	2.1957	2.0912
P3, Slovakia	1.8553	1.8195	2.0715	2.1657	1.9167
	2.2053	2.052	2.1527	1.8593	2.0819
	2.0415	1.9045	2.0403	2.098	2.0105
	2.0678	1.9951	1.9211	1.9968	2.0214
	2.0376	1.9811	2.1122	1.9778	2.1271

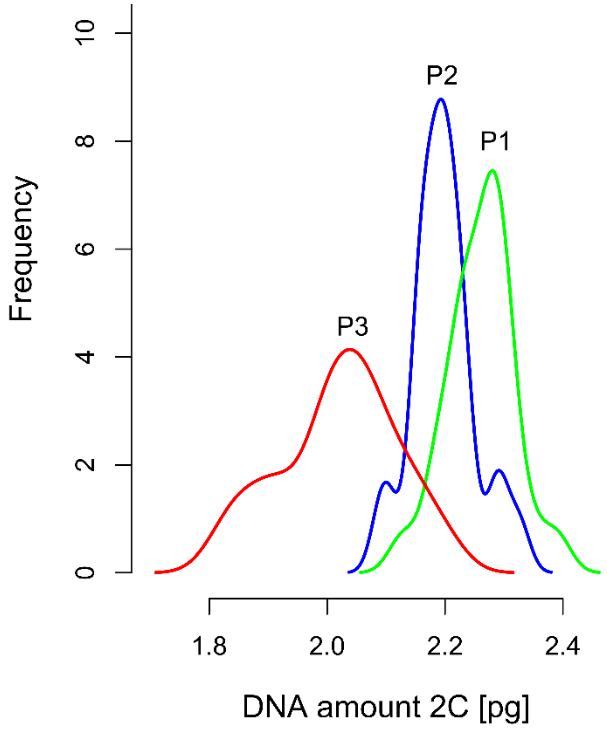

Supplementary Fig. 1. Histograms calculated from Supplementary Table 2.

Linear mixed effects models

Supplementary Table 3. Linear mixed effects model coefficients and statistics for the DNA amount of ragweed (two genome size groups are not distinguished), and meteorological and geographical variables as fixed effects, and an individual plant as a random effect.

Variables (fixed effects)		DNA amount (intercept)	beta	SE	t (p-value) df = 370	R^2c	R^2m
T_{mean}	■	1.645	0.0496	0.0049	10.17***	0.997	0.358
	□	1.626	0.046	0.0046	9.88***	0.997	0.345
T_{mean} May–October	■	1.409	0.0439	0.0045	9.73***	0.997	0.339
	□	1.400	0.0412	0.0043	9.55***	0.997	0.330
T_{mean} December–March	■	2.061	0.059	0.005	10.95***	0.997	0.393
	□	2.034	0.054	0.005	10.4***	0.997	0.369
P	■	2.468	-0.0006	0.00006	-9.33***	0.997	0.320
	□	2.444	-0.0005	0.00006	-7.47***	0.997	0.232
P May–October	■	2.416	-0.0008	0.00008	-9.41***	0.997	0.324
	□	2.406	-0.0007	0.0001	-7.08***	0.997	0.213
P December–March	■	2.429	-0.002	0.0003	-7.16***	0.997	0.217
	□	2.315	-0.001	0.0003	-4.58***	0.997	0.102
Altitude		2.18	-0.0003	0.00003	-8.41***	0.997	0.291
Latitude		12.5	-0.215	0.02	-10.7***	0.997	0.384
Longitude		2.879	-0.041	0.006	-6.99***	0.997	0.209

*p < 0.05, **p < 0.01, ***p < 0.001; T_{mean} – mean air temperature, P total precipitation, ■ historical period (1970–2000), □ current period (2020–2021); SE = Standard error, R^2c – conditional R^2 for random and fixed effects, R^2m – marginal R^2 for fixed effects

Supplementary Table 4. Linear mixed effects model coefficients and statistics for the DNA amount of ragweed (two genome size groups are not distinguished), and meteorological and geographical variables as fixed effects, and an individual plant as a random effect. The outliers (2C < 1.185 pg and 2C > 2.4 pg) were excluded.

Variables (fixed effects)		DNA amount (intercept)	beta	SE	t (p-value) df = 177	R^2c	R^2m
T_{mean}	■	1.657	0.048	0.005	10.17***	0.997	0.325
	□	1.650	0.044	0.005	9.17***	0.997	0.315
T_{mean} May–October	■	1.434	0.042	0.0047	8.96***	0.997	0.301
	□	1.439	0.039	0.0044	8.78***	0.997	0.296
T_{mean} December–March	■	2.060	0.057	0.006	10.34***	0.997	0.367
	□	2.036	0.051	0.005	9.79***	0.997	0.344
P	■	2.433	-0.0005	0.00006	-8.24***	0.997	0.271
	□	2.405	-0.0004	0.00007	-6.55***	0.997	0.193
P May–October	■	2.387	-0.0007	0.00009	-8.36***	0.997	0.275
	□	2.369	-0.0006	0.0001	-6.14***	0.997	0.173
P December–March	■	2.381	-0.002	0.0003	-7.16***	0.997	0.173
	□	2.277	-0.001	0.0003	-3.87***	0.997	0.077
Altitude		2.174	-0.0003	0.00003	-8***	0.997	0.255
Latitude		11.54	-0.195	0.02	-10.2***	0.997	0.364
Longitude		2.808	-0.037	0.005	-6.8***	0.997	0.205

*p < 0.05, **p < 0.01, ***p < 0.001; T_{mean} – mean air temperature, P total precipitation, ■ historical period (1970–2000), □ current period (2020–2021); SE = Standard error, R^2c – conditional R^2 for random and fixed effects, R^2m – marginal R^2 for fixed effects

Supplementary Table 5. Linear mixed effects model coefficients and statistics for the DNA amount of ragweed (two genome size groups are not distinguished), and meteorological and geographical variables as fixed effects, and a population as a random effect

Variables (fixed effects)		DNA amount (intercept)	beta	SE	t (p-value) df = 35	R^2c	R^2m
T_{mean}	■	1.645	0.0496	0.009	5.48***	0.771	0.352
	□	1.626	0.046	0.0087	5.29***	0.771	0.339
T_{mean} May–October	■	1.409	0.0439	0.0084	5.2***	0.771	0.333
	□	1.400	0.0412	0.0081	5.08***	0.771	0.324
T_{mean} December–March	■	2.061	0.059	0.0098	5.99***	0.771	0.387
	□	2.034	0.054	0.0095	5.61***	0.771	0.363
P	■	2.468	-0.0006	0.0001	-4.95***	0.771	0.314
	□	2.444	-0.0005	0.0001	-3.86***	0.773	0.227
P May–October	■	2.416	-0.0008	0.00015	-5***	0.771	0.318
	□	2.406	-0.0007	0.0002	-3.64***	0.773	0.208
P December–March	■	2.429	-0.002	0.0003	-3.69***	0.773	0.212
	□	2.315	-0.001	0.0006	-2.3*	0.774	0.099
Altitude		2.18	-0.0003	0.00006	-4.58***	0.772	0.285
Latitude		12.5	-0.215	0.036	-5.84***	0.771	0.378
Longitude		2.879	-0.041	0.011	-3.59***	0.773	0.204

*p < 0.05, **p < 0.01, ***p < 0.001; T_{mean} – mean air temperature, P total precipitation, ■ historical period (1970–2000), □ current period (2020–2021); SE = Standard error, R^2c – conditional R^2 for random and fixed effects, R^2m – marginal R^2 for fixed effects

Supplementary Table 6. Linear mixed effects model coefficients and statistics for the DNA amount of ragweed (two genome size groups are not distinguished), and meteorological and geographical variables as fixed effects, and a measurement date as a random effect

Variables (fixed effects)		DNA amount (intercept)	beta	SE	t (p-value) df = 527	R^2c	R^2m
T_{mean}	■	1.576	0.056	0.003	19.4***	0.510	0.420
	□	1.537	0.054	0.003	19.4***	0.523	0.420
T_{mean} May–October	■	1.293	0.051	0.003	18.8***	0.502	0.407
	□	1.259	0.049	0.003	18.8***	0.515	0.409
T_{mean} December–March	■	2.056	0.064	0.003	20.3***	0.510	0.438
	□	2.023	0.061	0.003	19.9***	0.518	0.429
P	■	2.525	-0.0006	0.00004	-17.7***	0.472	0.386
	□	2.516	-0.0006	0.00004	-14.5***	0.403	0.306
P May–October	■	2.465	-0.0009	0.00005	-17.9***	0.478	0.390
	□	2.482	-0.0009	0.00006	-14.2***	0.404	0.297
P December–March	■	2.468	-0.002	0.0002	-13.1***	0.325	0.259
	□	2.333	-0.001	0.0002	-8.17***	0.173	0.121
Altitude		2.187	-0.0003	0.00002	-16.4***	0.430	0.348
Latitude		12.8	-0.221	0.012	-18.7***	0.430	0.397
Longitude		2.889	-0.041	0.003	-11. 9***	0.241	0.214

*p < 0.05, **p < 0.01, ***p < 0.001; T_{mean} – mean air temperature, P total precipitation, ■ historical period (1970–2000), □ current period (2020–2021); SE = Standard error, R^2c – conditional R^2 for random and fixed effects, R^2m – marginal R^2 for fixed effects

DNA amount estimation from herbarium specimens

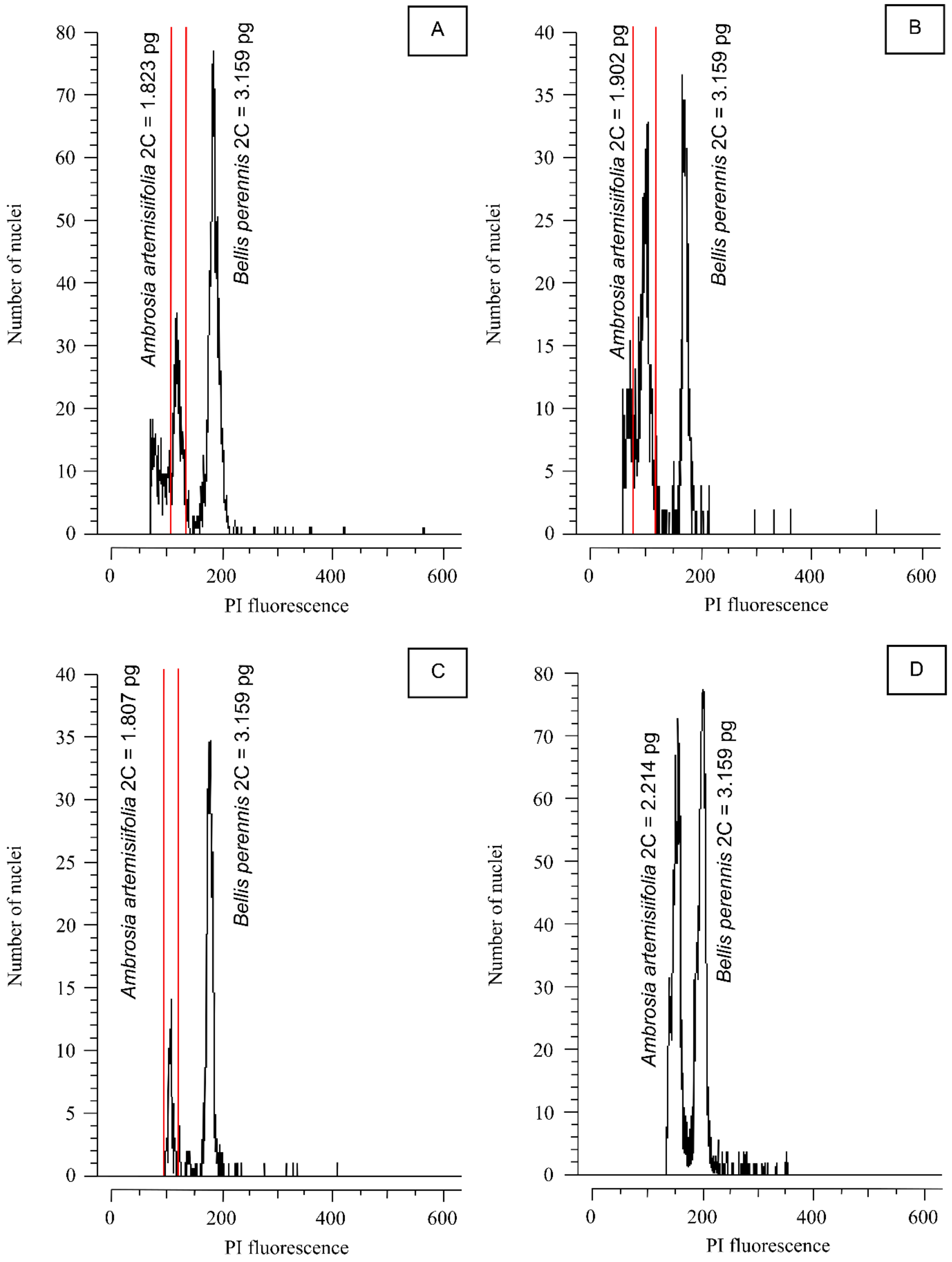

Supplementary Fig. 2. The flow cytometry histograms from herbarium specimen measurements. A. *Ambrosia artemisiifolia* collected in Komárno, 1949 by J. Futák; B.

Ambrosia artemisiifolia collected in Vráble, 1949 by J. Futák; C. *Ambrosia artemisiifolia* collected in Malacky, 1956 by J. Májovský. D. *Ambrosia artemisiifolia* collected in Rusovce, 1971 by B. Votavová. As standard, *Bellis perennis* was used (2C = 3.159 pg). For ragweed peak identification, a "gating" (red lines) was used. The estimated DNA amount can only be informative as not all peaks have the same size, low CV, or adequate nuclei number.

Ragweed genome size group and environmental variables

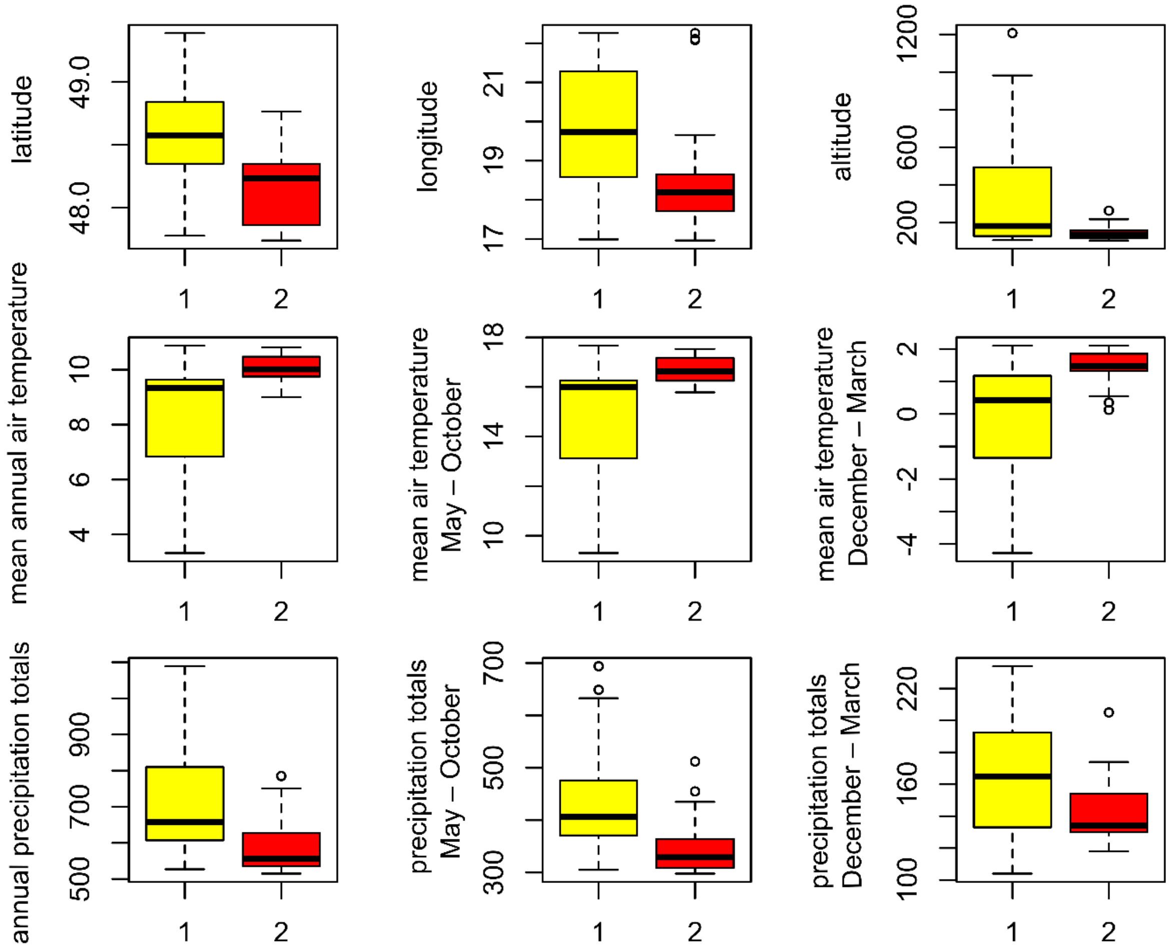

Supplementary Fig. 3. Boxplots showing relationships between the environmental factors (meteorological variables are from the historical period) and ragweed genome size groups in the study area; yellow – group 1 (2C < 2.1 pg), red – group 2 (2C > 2.1 pg).

SDM of genome size distribution

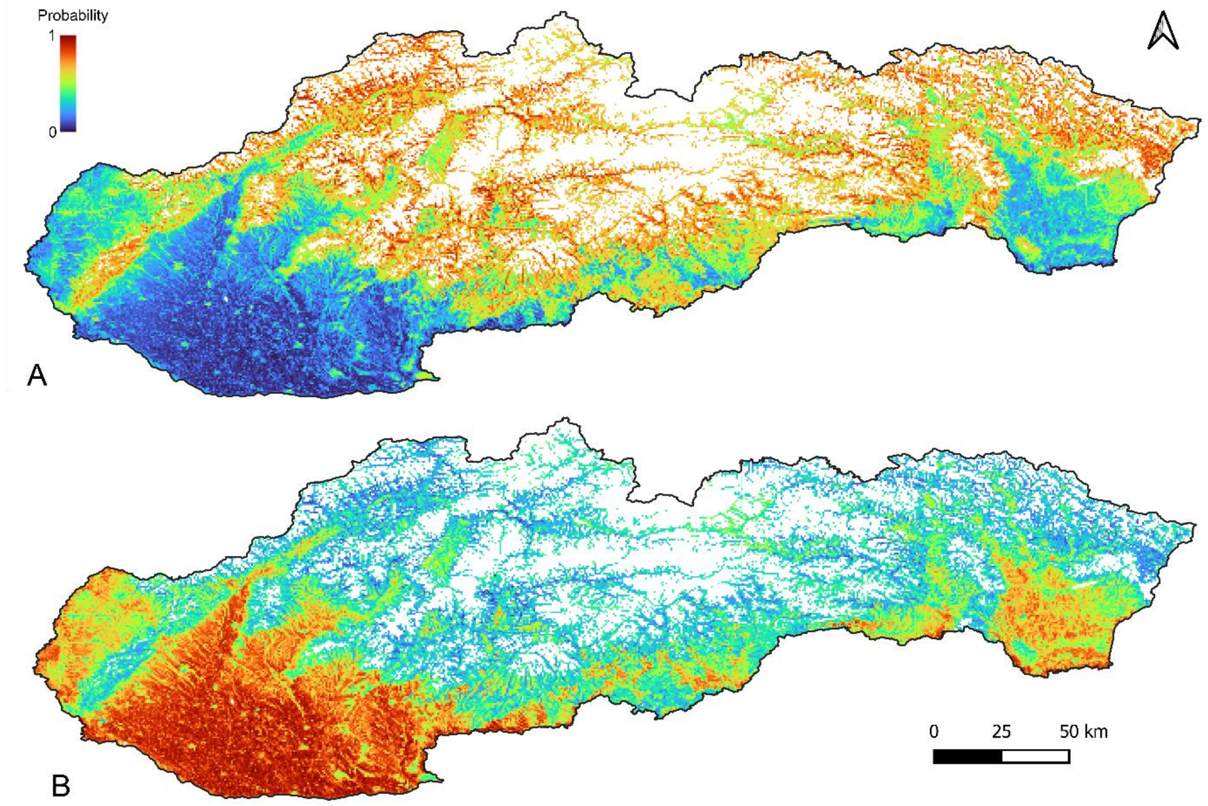

Supplementary Fig. 4. Genome size distribution models. The model A predicts the probability of the occurrence of group 1 (2C < 2.1 pg) in the study area. The probability is higher in mountainous regions and greater latitudes. The model B shows the probability of the occurrence of group 2 (2C > 2.1 pg), which is higher in lowlands and basins with the higher mean annual air temperature. The reliability of both models is higher than 70%.
